# Interrater reliability of different scoring systems for drug-induced sleep endoscopy

**DOI:** 10.1007/s11325-024-03190-2

**Published:** 2024-11-29

**Authors:** Dimitrios Mitsikas, Benedikt Jakob, Vlado Janjic, Corinne Hasler, Samuel Tschopp

**Affiliations:** 1 Department of Otorhinolaryngology, Head and Neck Surgery, Kantonsspital Baselland, Liestal, Switzerland; 2 Department of Otorhinolaryngology, Head and Neck Surgery, Inselspital, University Hospital and University of Bern, Freiburgstrasse 20, Bern, 3010 Switzerland

**Keywords:** Drug-induced sleep endoscopy, DISE, Obstructive sleep apnea, Interrater reliability, VOTE classification, PTLTbE classification

## Abstract

**Purpose:**

To explore the interobserver reliability of drug-induced sleep endoscopy (DISE) for patients with obstructive sleep apnea syndrome (OSAS) of two classification systems.

**Methods:**

DISE examinations were recorded digitally for all patients and were evaluated independently by five examiners blinded to all patient data. Areas of obstruction were rated using VOTE (velum, oropharynx lateral wall, tongue base, epiglottis) classification and PTLTbE (palate, tonsils, lateral pharyngeal wall, tongue base, epiglottis) classification. Additionally, palatal coupling was assessed during the jaw thrust maneuver. Interobserver reliability was evaluated with Fleiss’ kappa for categorical data and linearly weighted kappa for ordinal data.

**Results:**

In total, 123 patients were included in this study, 92 males and 31 females, with a mean (SD) age of 50.7 (12.1) years and a body mass index (BMI) of 28.3 (5.0) kg/m2. The mean apnea-hypopnea index was 22.2 (18) per hour, and the Epworth Sleepiness Scale was 7.3 (2.3). In our cohort, the interobserver reliability for the VOTE classification ranged from 0.32 to 0.59 and for the PTLTbE classification from 0.23 to 0.49 over all assessed levels, indicating fair to moderate interobserver reliability. The weighted kappa for palatal coupling was 0.37. In the VOTE classification, the level with the best agreement was the velum, while in PTLTbE, the best agreement was observed for tonsillar obstruction.

**Conclusion:**

The interrater agreement was fair to moderate for both classifications, with no clear superiority for one classification. The large variability shows the need to objectively quantify upper airway collapse during DISE and natural sleep endoscopy.

## Introduction

Although polysomnography is the gold standard diagnostic study for sleep-disordered breathing, it fails to establish the anatomic location of upper airway obstructions [1]. Knowing the exact location of the obstruction is critical for successful sleep surgery or other site-specific interventions, such as oral appliances [2, 3]. Studies suggest that DISE, when compared to awake assessments, changes the surgical treatment plan in approximately 50% of patients with obstructive sleep apnea (OSA) [4]. There is also evidence that the success rate of surgical treatment of OSA in patients selected by DISE is higher than in patients not undergoing DISE before surgical treatment [5]. Drug-induced sleep endoscopy (DISE) uses a flexible nasopharyngoscope to visualize and assess the upper airway under sedation to determine the level, degree, and configuration of collapse in patients with sleep-disordered breathing [6]. During DISE, the upper airway is examined, and levels of obstruction are noted. Several different classification systems have been described to standardize the description of examination findings. However, no classification is universally accepted [7]. The VOTE classification is the most used system [8]. More recently, the PTLTbE classification has been published, simplifying certain levels and introducing a distinction between tonsil and lateral pharyngeal wall collapse [9].

Despite the usefulness of DISE in providing information on the site and the pattern of airway collapse, only a few studies have assessed the reliability of DISE. Test-retest reliability between separate DISE examinations has been shown to be good, and a good agreement exists between DISE and natural sleep [10, 11]. However, the results of DISE might vary between observers (interobserver reliability) or even within an individual observer (intraobserver reliability) [12–14]. Currently, no studies are comparing the interobserver reliability between different classification systems of DISE. This study aims to compare the interobserver reliability of DISE using the VOTE [8] and PTLTbE [9] classifications for patients with OSA.

## Materials and methods

A retrospective analysis of the data collected at our center between July 2021 and December 2021 was performed. This retrospective cohort study was conducted according to the Declaration of Helsinki and approved by the Swiss ethics committee (ΕΚΝΖ 2021–02321). Patients were offered DISE based on history, physical examination, and respiratory polygraphy. Inclusion criteria included apnea-hypopnea index ≥ 5/h on a sleep study. Patients were excluded if they were under 18 years old, no informed consent was given, or medical data regarding the primary endpoints was missing.

### Baseline assessment

Each patient was clinically evaluated preoperatively using a standardized report form. Baseline characteristics were recorded, including gender, age, height, weight, body mass index (BMI), and neck circumference. The Epworth Sleepiness Scale was used to assess daytime sleepiness. Home apnea sleep testing was performed using a respiratory polygraphy.

### Drug-induced sleep endoscopy

All subjects underwent DISE in the operating room according to the recommendations by the European position paper [7]. DISE was performed in a dark and silent operating room with the patient lying supine. No anticholinergic or topical anesthesia of the nose was used during the procedure. Sedation was introduced using a single intravenous dose of midazolam (bolus injection of 2 mg) and maintained with propofol by target control infusion pump with a starting dose of 1.5 mcg/mL. If required, the rate was increased by 0.2-0.5 mcg/mL every 2 min until a stable sedation was achieved. The sedation level was recorded using a bispectral index monitor (BIS). Patients were monitored throughout the examination using electrocardiography, pulse oximetry, and non-invasive blood pressure measurements. Supplementary oxygen was not used routinely but was administered if the patient’s oxygen saturation dropped below 90%. When the patient was asleep and actively snoring with BIS between 50 and 70, a video-recorded fiberoptic nasopharyngoscope was used to assess the upper airway. After examination of each level for at least two cycles of obstructions, a jaw thrust maneuver was performed to evaluate the palatal coupling.

### Blinded rating using the VOTE and PTLTbE classifications

DISE was recorded digitally for all patients, and five blinded examiners evaluated the video recordings independently. All examiners were experienced in performing and scoring DISE. Areas of obstruction were evaluated using VOTE and PTLTbE classification [8, 9]. The VOTE classification assesses the upper airway obstruction at four anatomical levels: velum, oropharynx, tongue, and epiglottis. The degree of obstruction can be classified as 0, indicating no obstruction; 1 for partial obstruction or vibrations; and 2 for complete obstruction. The obstruction configuration at the velum level is categorized as anteroposterior, lateral, or concentric. Oropharyngeal obstruction occurs only in the lateral direction. Obstruction at the tongue base occurs only in the anteroposterior configuration. Epiglottis obstruction can be classified in anteroposterior and lateral configurations [8].

The PTLTbE classification assesses the upper airway obstruction at five anatomical levels: palate, tonsils, lateral pharyngeal wall, base of the tongue, and epiglottis. The palatal obstruction can be described as P0, indicating no obstruction; P1, indicating anteroposterior collapse; and P2 for concentric collapse. The tonsillar obstruction is graded as T0 for no obstruction, T1 for less than 50% obstruction, and T2 for greater than 50% obstruction. Lateral pharyngeal wall obstruction is categorized analogously to the tonsillar obstruction in L0, L1, and L2. The tongue base collapses posteriorly. Tb0 is when the tongue base does not touch the epiglottis. Tb1 is defined as less than 50% obstruction, and Tb2 is defined as more than 50% obstruction. Epiglottic obstruction is described as E0 for no obstruction and E1 for a trap door phenomenon [9].

In addition to the VOTE and PTLTbE classification, palatal coupling was also analyzed. This indicates the opening of the upper airway during jaw thrust. The degree of opening was classified as 0 for no change of the obstruction, 1 for partial opening, and 2 for complete opening of the upper airway.

### Statistical analysis

All statistical analyses were performed using R version 4.3.3 (R Foundation for Statistical Computing, Vienna, Austria). Descriptive statistics were used to analyze patient characteristics. The interobserver reliability was assessed using the Fleiss’ kappa. For ordinal data, such as the degree of collapse, linear weights were introduced based on the assumption of equal spacing between near-matches [15, 16]. To assess the effect of experience, the variability between the two most experienced raters was compared to the variability of the two least experienced raters. For the interobserver reliability, the following categories were accepted: poor (kappa < 0.20), fair (kappa: 0.21–0.40), moderate (kappa: 0.41–0.60), good (kappa: 0.61–0.80), excellent (kappa: >0.81) [17]. To assess the influence of sedation depth on collapse pattern, the collapse grade ratings were modeled using a mixed-effects model with a random effect for each patient and a fixed effect for sedation depth. For the collapse configuration, we used Kruskal-Wallis tests. Results with a P value below 0.05 were considered statistically significant.

## Results

### Patients’ characteristics

Consecutive DISE recordings of 132 patients between July and December 2021 have been retrieved. Nine patients were excluded for insufficient recording quality or missing video. In total, 123 patients were included in this study, 92 males and 31 females, with a mean (SD) age of 50.7 (12.1) years and a BMI of 28.3 (5.0) kg/m2. The bispectral index at the beginning of the DISE was 58.6 (8.9). All patient characteristics are summarized in Table 1. All 123 patient recordings were scored by five blinded raters, resulting in a total of 615 ratings for analysis. An overview of all scorings for the VOTE and PTLTbE classifications by all raters is given in Table 2. Most patients displayed multilevel collapse. Palatal obstruction was most frequently observed in both classifications. An epiglottic collapse was frequently scored in the VOTE classification, mainly in an anterior-posterior direction, while only 29% scored an epiglottic collapse pattern using the PTLTbE classification. Most patients showed at least a moderate palatal coupling during the jaw thrust, and in 44% of patients, the upper airway obstruction was resolved entirely.

### Interrater reliability

**Fig. 1:**
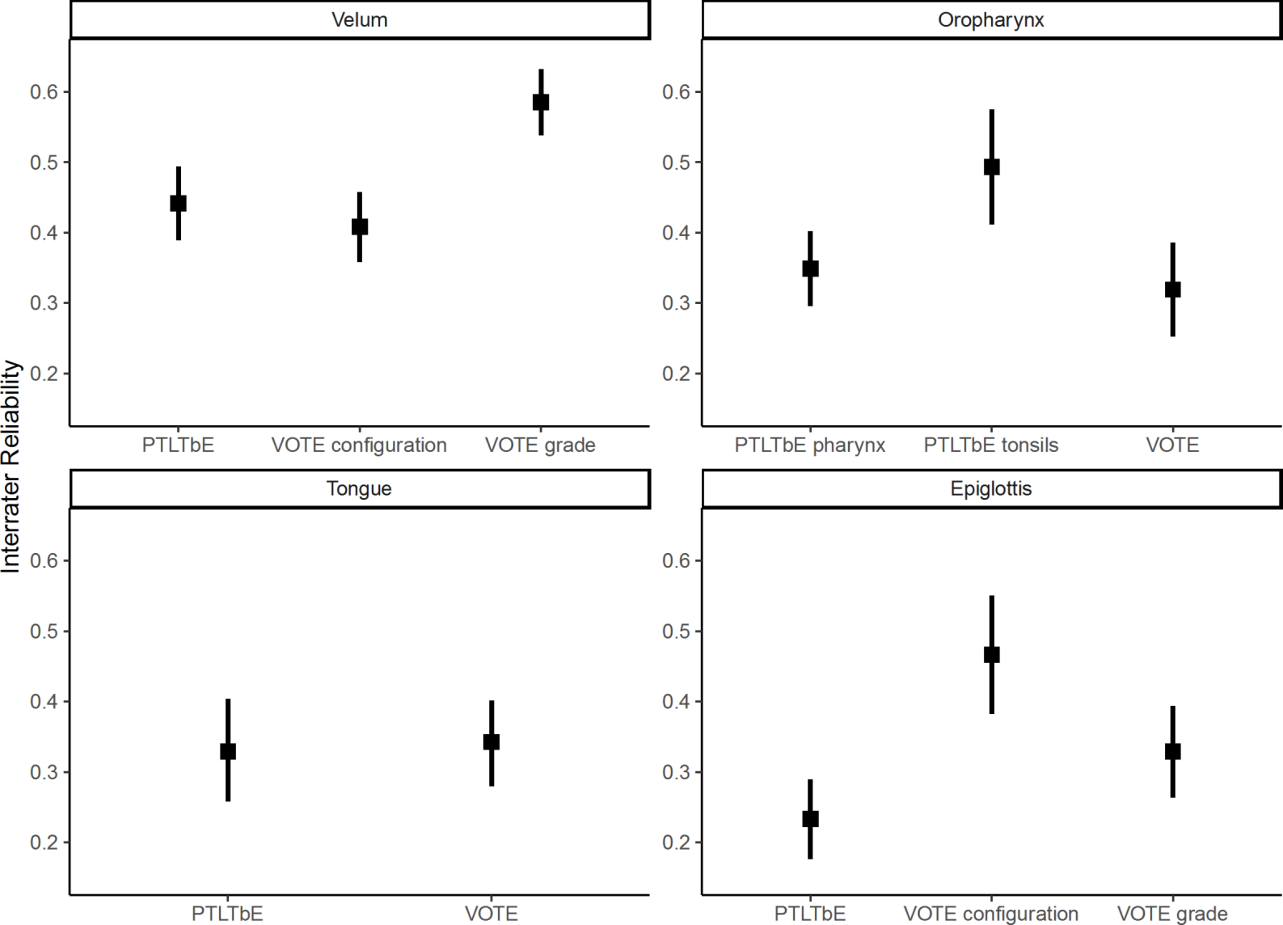
Interrater agreement by anatomical level and classification system. Rectangles indicate the Fleiss’ kappa, with error bars representing the 95% confidence interval. For nominal data, such as the grades of collapse, a linear weighted Fleiss’ kappa was calculated

An overview of the interrater agreement for all levels with their respective grades and configurations is given in Table 3 for the VOTE and Table 4 for the PTLTbE classifications. In our cohort, the interobserver reliability for the VOTE classification ranged from 0.32 to 0.59, and the PTLTbE classification from 0.23 to 0.49, indicating a fair to moderate interobserver agreement [17]. The interrater reliability for the palatal coupling is fair, with 0.37 (95% CI, 0.31–0.43). The best agreement has been observed for the collapse grade of the velum in the VOTE classification. Only moderate agreement exists for the configuration of velar collapse configuration. The interrater reliability is visualized in four anatomical levels in Fig. 1, illustrating that no classification system is superior.

To analyze the effect of experience on the interrater variability, we compared the variability between the two most experienced raters with that of the two least experienced examiners (Table 5). We found no overall difference between the experienced and inexperienced raters, indicating no effect of experience on the reliability. However, the agreement among experienced raters was lower for the epiglottis in both classifications than less experienced raters. The experienced raters agreed more about the tongue base collapse in the VOTE classification than inexperienced examiners. The influence of sedation depth measured by the BIS was calculated for the collapse degree and configuration (Table 6). Using the VOTE classification, significantly lower sedation was observed for the anterior-posterior collapse of the velum and higher collapse degree of the epiglottis. No effect of BIS on the PTLTbE was observed.

## Discussion

DISE is a valuable tool in assessing the upper airway collapse site for site-specific treatment of sleep-disordered breathing. DISE changes the surgical treatment plan in about 50% of patients with OSA [4], and there is evidence that the success rate of surgical treatment is higher in patients selected by DISE [5].

A meta-analysis showed that more than 15 classification systems have been published to assess DISE [18]. However, no classification system is universally accepted and can be used as the gold standard [7]. The VOTE classification is the most widely used system [8]. The PTLTbE classification has more recently been described, and the authors find the simplification of the velar collapse pattern and the distinction between the lateral pharynx and tonsillar collapse clinically useful [9]. Therefore, we focused our interrater reliability analysis on these two systems.

The test-retest reliability and the agreement of DISE with natural sleep are supported by several studies [10, 11, 19]. However, interobserver agreements range from poor to good for classifications in the literature. The VOTE classification has been most extensively studied. The inventors of the VOTE classification, Kezirian et al., describe moderate interrater reliability [13]. Altintas et al. included 55 patients and compared the consistency of three different observers. They reported an overall interobserver agreement between poor and good. The level with the best agreement was the soft palate [20]. Carrasco-Llatas et al. analyzed 31 patients rated by two examiners and found poor to good agreement depending on the level of obstruction. The highest percentage of agreement was reported at the oropharynx level, and poor agreement for the palate [12].

In our study, interobserver reliability for both classifications was fair to moderate. The level with the best agreement was the collapse grade of the velum, but it had relatively low reliability on its configuration. In literature, there seems not to be a region with clearly lower variability. In our opinion, variability is multifactorial. The natural variability between raters should affect the variability of all areas equally. However, throughout a DISE, several factors, such as sedation depth, might change slightly. Certain regions may be affected by this to a larger degree than others, resulting in a larger variability, depending on which timeframe is estimated as representative by an observer. Generally, the lack of greater agreement suggests that the classification for both systems is often interpreted subjectively. This subjectivity may be related to many factors, such as the observer’s experience.

Vroegop et al. assessed the inter- and intrarater consistency in both experienced and inexperienced groups of observers using 6 DISE videos and 97 observers. They concluded that both interrater and intrarater consistencies were higher for experienced observers than inexperienced observers, demonstrating the importance of experience in assessing DISE [14]. In our data, we could not observe any systematic differences depending on the experience. Since none of our raters were at least moderately experienced, this finding should be interpreted cautiously. Similarly, the observation of more concentric velum collapse and less epiglottis collapse in the VOTE classification must be treated cautiously. Our data suggest that the interobserver agreement for the VOTE classification is fair to moderate, which is in line with previous results. The PTLTbE classification was introduced in 2020 by Veer et al., and the interobserver reliability was tested using ratings of 20 doctors of 5 DISE videos. They reported moderate to good interobserver agreement [9]. Our data suggest that the interobserver agreement for the PTLTbE classification is lower.

We acknowledge the potential limitations of our study. In our study, blinded video recordings were examined. During the recording, additional information on the examination might be lost, increasing the variability of the ratings. Further, our patient collective is primarily male, middle-aged, and moderately overweight. This limits generalizability to other patient groups. We used Fleiss’ kappa to estimate the interrater agreement, which is heavily dependent on the observed marginal frequencies. Some collapse patterns are more rarely observed, and thus, comparison between levels is limited.

The strengths of this study are the large number of ratings. To our knowledge, this is the most extensive study on interrater variability of DISE. Further, the DISE procedure is standardized in our institution, and all examiners perform the same maneuvers. All DISE examinations are recorded, and only a few had to be excluded from the analysis, reducing bias.

Future research should aim to quantify the upper airway collapse pattern objectively. Several attempts have been made to use computer assistance or artificial intelligence to analyze DISE recordings quantitatively [21, 22].

## Conclusion

Several classification systems exist to describe upper airway collapse during DISE. We analyzed the interobserver reliability for the VOTE and PTLTbE classifications. In our study, the interrater reliability of both systems was fair to moderate, highlighting the subjective interpretation of DISE results. An objective evaluation of the upper airway collapse during DISE should provide reliable results independent of the examiner and standardize the examination. Future research is warranted to objectively quantify the upper airway anatomy during DISE to improve the reliability of the examination, irrespective of the classification system.

**Table 1:** Characteristics of study participants are given as mean and standard deviation or number and percentage

Characteristic	Mean (SD)
Total Patients (No)	123
males No. (%)	92 (74.8)
females No. (%)	31 (25.2)
Age (years)	50.7 (12.1)
Height (cm)	175.7 (8.8)
Weight (kg)	97.5 (17)
Neck circumference (cm)	41 (3.7)
Body mass index (kg/m2)	28.3 (5)
Epworth sleepiness scale	7.3 (2.3)
Snoring index (VAS 0–10)	6.9 (4.7)
Sleep apnea testing	
Apnea-hypopnea index (events/hour)	22.2 (18)
Oxygen desaturation index (events/hour)	12.6 (13.4)
Mean oxygen saturation (%)	93.6 (1.4)
Time below 90% oxygen saturation (% of sleep)	3.1 (8.3)
Heart rate (beats per minute)	62.3 (8.9)

**Table 2:** Overview of collapse patterns assessed according to the VOTE and PTLTbE classification by the five blinded reviewers

	Grade (% and *n*)			Configuration (% and *n*)		
	0	1	2	anterior-posterior	lateral	concentric
**VOTE**						
Velum	3% (19)	41% (249)	56% (346)	48% (290)	6% (38)	46% (277)
Oropharynx lateral walls	40% (243)	43% (262)	18% (109)			
Tongue Base	25% (154)	48% (292)	27% (167)			
Epiglottis	22% (132)	41% (250)	38% (231)	92% (473)	8% (43)	
**PTLTbE**						
Palate				3% (17)	48% (297)	49% (299)
Tonsils	70% (427)	18% (108)	13% (79)			
Lateral pharyngeal walls	29% (177)	51% (310)	21% (126)			
Tongue Base	24% (146)	35% (217)	41% (250)			
Epiglottis	71% (432)	29% (180)				
Palatal Coupling	10% (59)	47% (285)	44% (266)			

Grades for the VOTE classification were defined as 0 = no obstruction, 1 = partial obstruction or fluttering, and 2 = complete obstruction. For the PTLTbE, the categories are 0 = no obstruction, 1 = below 50% obstruction, and 2 = above 50% obstruction. The palatal coupling was graded into 0 = no coupling, 1 = mild opening with some residual collapse, and 2 = no collapse after the jaw thrust maneuver

**Table 3:** Interrater reliability for the VOTE classification

Level	Grade		Configuration	
	Raw Agreement (%)	Fleiss’ kappa (95% CI)^a^	Raw Agreement (%)	Fleiss’ kappa (95% CI)^a^
Velum	63	0.59 (0.54–0.63)	67	0.41 (0.36–0.46)
Oropharynx lateral walls	47	0.32 (0.26–0.38)	-	-
Tongue Base	48	0.34 (0.28–0.41)	-	-
Epiglottis	48	0.33 (0.27–0.4)	91	0.47 (0.38–0.55)

^a^ The Fleiss’ kappa was calculated for categorical data (e.g., configuration) and with linear weights for ordinal data (e.g., grade)

**Table 4:** Interrater reliability for the PTLTbE classification

Level	Grade		Configuration	
	Raw Agreement (%)	Fleiss’ kappa (95% CI)^a^	Raw Agreement (%)	Fleiss’ kappa (95% CI)^a^
Palate	-	-	71	0.44 (0.39–0.49)
Tonsils	63	0.49 (0.41–0.58))	-	-
Lateral pharyngeal wall	47	0.35 (0.3–0.4)	-	-
Tongue Base	50	0.33 (0.26–0.4)	-	-
Epiglottis	68	0.23 (0.18–0.29)	-	-

^a^ The Fleiss’ kappa was calculated for categorical data (e.g., configuration) and with linear weights for ordinal data (e.g., grade)

**Table 5:** Interrater reliability depending on experience

	Experienced Rater ^a^		Inexperienced Rater ^a^	
	Raw Agreement (%)	Kappa (95% CI)	Raw Agreement (%)	Kappa (95% CI)
**VOTE**				
Velum Grade	67	0.65 (0.54–0.74)	61	0.57 (0.47–0.67)
Velum Configuration	73	0.51 (0.35–0.66)	65	0.4 (0.26–0.54)
Oropharynx Lateral Walls	62	0.54 (0.45–0.64)	31	-0.03 (-0.18–0.13)
Tongue Base	46	0.36 (0.25–0.47)	33	0.12 (0–0.26)
Epiglottis Grade	55	0.44 (0.32–0.55)	45	0.33 (0.21–0.45)
Epiglottis Configuration	86	0.16 (-0.04–0.36)	92	0.53 (0.35–0.71)
**PTLTbE**				
Palate	77	0.56 (0.4–0.72)	70	0.42 (0.26–0.58)
Tonsils	63	0.49 (0.34–0.61)	72	0.66 (0.54–0.77)
Lateral pharyngeal walls	54	0.47 (0.35–0.57)	46	0.31 (0.18–0.46)
Tongue Base	44	0.25 (0.13–0.37)	40	0.21 (0.09–0.35)
Epiglottis	56	0.08 (-0.09–0.26)	73	0.34 (0.16–0.52)
**Palatal Coupling**	54	0.45 (0.35–0.56)	53	0.44 (0.32–0.56)

^a^ The interrater reliability between the two most experienced raters is compared to the reliability of the two least experienced raters

**Table 6:** Collapse pattern depending on sedation depth

	Grade		Configuration			
	Estimate (95% CI)	*p*-value	anterior-posterior	lateral	concentric	*p*-value
**VOTE**						
Velum	0.008 (-0.001–0.016)	0.06	57.5 (9.3)	59.6 (7.7)	59.8 (8.1)	0.03
Oropharynx lateral walls	0.0002 (-0.007–0.01)	0.67				
Tongue Base	-0.007 (-0.018–0.003)	0.17				
Epiglottis	-0.01 (-0.02 - -0.002)	0.02	58.2 (9.0)	56.4 (7.3)		0.11
**PTLTbE**						
Palate			56.3 (9.8)	57.8 (9.3)	59.6 (8.0)	0.10
Tonsils	0.002 (-0.008–0.01)	0.65				
Lateral pharyngeal walls	0.003 (0.006–0.01)	0.52				
Tongue Base	-0.01 (-0.02–0.001)	0.06				
Epiglottis						
**Palatal Coupling**	-0.007 (-0.01–0.002)	0.12				

The influence of sedation depth using the bispectral index on collapse grade ratings was modeled using a mixed-effects model with a random effect for each patient and a fixed effect for sedation depth. For the collapse configuration, the BIS values are given and compared using Kruskal-Wallis tests.

## Data Availability

The data that support the findings of this study are available on request from the corresponding author, Samuel Tschopp.
